# Gram-positive and gram-negative subcellular localization using rotation forest and physicochemical-based features

**DOI:** 10.1186/1471-2105-16-S4-S1

**Published:** 2015-02-23

**Authors:** Abdollah Dehzangi, Sohrab Sohrabi, Rhys Heffernan, Alok Sharma, James Lyons, Kuldip Paliwal, Abdul Sattar

**Affiliations:** 1 Institute for Integrated and Intelligent Systems (IIIS), Griffith University, Kessels Road, Brisbane, Australia; 2 National ICT Australia (NICTA), Kessels Road, Brisbane, Australia; 3 School of Engineering, Griffith University, Kessels Road, Brisbane, Australia; 4 School of Engineering, The University of the South Pacific, Suva, Fiji

**Keywords:** Protein Subcellular Localization, Gram-positive Bacterial Proteins, Gram-negative Bacterial Proteins, Feature Extraction, Segmentation-based Method, Physicochemical-based Features, Evolutionary-base Features, Rotation Forest

## Abstract

**Background:**

The functioning of a protein relies on its location in the cell. Therefore, predicting protein subcellular localization is an important step towards protein function prediction. Recent studies have shown that relying on Gene Ontology (GO) for feature extraction can improve the prediction performance. However, for newly sequenced proteins, the GO is not available. Therefore, for these cases, the prediction performance of GO based methods degrade significantly.

**Results:**

In this study, we develop a method to effectively employ physicochemical and evolutionary-based information in the protein sequence. To do this, we propose segmentation based feature extraction method to explore potential discriminatory information based on physicochemical properties of the amino acids to tackle Gram-positive and Gram-negative subcellular localization. We explore our proposed feature extraction techniques using 10 attributes that have been experimentally selected among a wide range of physicochemical attributes. Finally by applying the Rotation Forest classification technique to our extracted features, we enhance Gram-positive and Gram-negative subcellular localization accuracies up to 3.4% better than previous studies which used GO for feature extraction.

**Conclusion:**

By proposing segmentation based feature extraction method to explore potential discriminatory information based on physicochemical properties of the amino acids as well as using Rotation Forest classification technique, we are able to enhance the Gram-positive and Gram-negative subcellular localization prediction accuracies, significantly.

## Introduction

Bacterial proteins are considered to be among the most important proteins and play a wide range of both useful and harmful roles. They are categorized as *Prokaryotic *microorganisms and generally can be divided into two groups namely: Gram-positive and Gram-negative [1]. The main difference between these two groups is that Gram-positive bacterial proteins have ticker cell wall containing many layers (consists of peptidoglycan and teichoic acids) while Gram-negative bacterial proteins have a tinner cell wall containing of a few layers (consists of only peptidoglycan). This causes difference between Gram-positive, and Gram-negative bacteria in reaction to antibiotics. In fact, despite ticker cell wall, Gram-negative are more resistent to antibiotics than Gram-positive bacteria due to their impenetrable lipid layer in their outer membrane [2]. The importance of bacteria, regardless of being Gram-positive and Gram-negative, is because they are the active elements on many useful biological interactions and at the same time, they are the source of many diseases which makes it crucially important to determine their functions especially for drug and vaccine design [3].

To be able to function properly, a protein (including the Gram-positive and Gram-negative bacterial proteins) needs to be in its appropriate subcellular place. Given a protein, determining its functioning place in the cell is called protein subcellular localization which is a difficult problem for computational biology and bioinformatics. Especially knowing that some proteins can function in more than one subcellular location which turn it to multi-label problem. This problem can be defined as a multi-class classification task in pattern recognition where its performance relies on the discriminatory information embedded in the extracted features as well as the performance of the classification technique being used.

Since the introduction of the protein subcellular localization problem in [4], a wide range of classification techniques have been used to tackle this problem [2,5,6]. Among the employed classifiers, the best results achieved by using *Support Vector Machine (SVM) *[7], *Artificial Neural Network (ANN) *[8], and *K-Nearest Neighbor (KNN) *[9]. However, recent studies have shifted their focus to enhance protein subcellular localization relying on better feature extraction techniques rather than exploring different classification techniques.

The early studies to tackle protein subcellular localization focused on sequence-based features to solve this problem [4]. Later on, wider range of features have been extracted to tackle this problem such as: physicochemical-based [6], Evolutionary-based [8], and Structural-based features [7]. However, the most significant enhancement for this task achieved by using *Gene Ontology (GO) *[10] information for feature extraction [11]. The term GO was coined to describe the properties of genes in organisms and its database established to represent molecular function, biological process and cellular components of proteins [8]. Despite its importance, GO has two main drawbacks. First, extracting GO for proteins produces a large number of features (over 18000 features) which needs further feature selection and filtering to extract adequate features [12]. Second, the GO information for new proteins is unavailable and many studies use homology-based approaches to extract GO for these proteins [13]. Hence, GO needs further investigation to be used as a reliable source for the feature extraction purposes.

In this study, we propose two overlapped segmentation-based feature extraction techniques to explore discriminatory information of physicochemical attributes of the amino acids. We investigate 117 different physicochemical attributes and select 10 best attributes for this task. We investigate our technique using the transformed protein sequences using evolutionary information embedded in the *Position Specific Scoring Matrix (PSSM) *to provide a mixture of physicochemical-based and evolutionary-based information. Finally, by applying the Rotation Forest classifier which to the-best-of-our-knowledge has not been explored previously for this task, we enhance the Gram-positive and Gram-negative subcellular localization prediction accuracies up to 3.4% compared to previous studies which have used GO for feature extraction. In this manner, we propose a new reliable method that explore the potential prediction ability of novel classification techniques as well as discriminatory information embedded in physicochemical and evolutionary-based features for protein subcellular localization.

## Data sets

In this study, we use two data sets that have been widely used in the literature for Gram-positive and Gram-negative subcellular localizations. For the Gram-positive subcellular localization, we use the data set that was proposed in [11,14,15]. This data set consists of 519 different proteins belonging to 4 Gram-positive subcellular locations. Among these 519 proteins, 515 belong to one location while 4 of these proteins belong to two locations. Hence, there are 523 (515 + 4 × 2) samples in this data set which are divided into four locations as follows: Cell membrane (174), Cell wall (18), cytoplasm (208), and Extracellular (123). This data set is publicly available at: http://www.csbio.sjtu.edu.cn/bioinf/Gpos-multi.

For the Gram-negative we have also used the data set that was introduced in [11,14,16]. This data set consists of 1392 different proteins belonging to 8 Gram-negative subcellular locations. Among these proteins 1328 belong to one location and 64 to two locations. Therefore, there are 1456 (1328 + 64 × 2) total samples in this data set which are divided into 8 locations as follows: Cell inner membrane (557), Cell outer membrane (124), Cytoplasm (410), Extracellular (133), Fimbrium (32), Flagellum (12), Nucleoid (8), and Periplasm (180). This data set is publicly available at: http://www.csbio.sjtu.edu.cn/bioinf/Gneg-multi/.

## Features

There are four feature groups extracted in this study in which two of them are physicochemical-based (overlapped segmented density and overlapped segmented autocorrelation) and two of them are evolutionary-based (semi composition and auto-covariance). To extract physicochemical-based feature groups, we first transform the protein sequence using evolutionary information and then extract physicochemical-based features from these transformed sequences [17]. We study 10 physicochemical attributes for feature extraction. These 10 attributes are selected among a wide range of physicochemical attributes in the following manner. First, we have extracted 117 physicochemical attributes from [18,19]. We then extract six feature groups based on each attribute using overlapped segmentation-based feature extraction techniques that have been explained in detail in [17]. In the next step, we have applied six different classifiers to each feature groups namely, *Naive Bayes*, KNN, SVM, *Multi-Layer Perceptron (MLP)*, *Multi-Class Adaptive Boosting (AdaBoost.M1)*, and *Random Forest*. Hence, we have 36 results (6 × 6) for a given attribute and 4212 results (36 × 117) for whole set of attributes for each data set (Gram-positive and Gram-negative). We then select 10 physicochemical attributes that individually attains the best results compared to the attributes (comparing the maximum, minimum, and average of all 36 results [20,21]). The experimental results for this step for both of our data sets are available upon request.

In this study, we aim at proposing novel feature extraction techniques to explore the potential discriminatory information of an individual physicochemical attribute of the amino acids. We have investigated these techniques for protein fold and structural class prediction problems and aim to investigate the generality of our proposed feature extraction techniques to capture local discriminatory information based on an individual physicochemical attribute of the amino acids [17,20,22]. We have also investigated the combinations of features extracted from a wider range of physicochemical attributes of the amino acids for protein fold and structural class prediction problems by using simplified segmentation-based feature extraction technique and will investigate these techniques for protein subcellular localization in our future works [23,24].

The list of selected features is as follows: (1) Average number of surrounding residues, (2) Polarity, (3) *Retardation Factor (RF) *chromatographic index, (4) Mean *Root Mean Square (RMS) *fluctuational displacement, (5) Solvent accessible reduction ratio, (6) Partition Coefficient, (7) Rigidity, (8) Average surrounding hydrophobicity, (9) Hydrophobicity scale (contact energy derived from 3D data), and (10) Hydrophilicity scale derived from *High-Performance Liquid Chromatography (HPLC) *peptide retention data. For the rest of this study, we will refer to these attributes by the number that are assigned to them as in Table 1.

**Table 1:** The list of the physicochemical attributes and the number assigned to them.

**No**.	Physicochemical Attirbutes
1	Average number of surrounding residues
2	Polarity
3	*Retardation Factor (RF) *chromatographic index
4	Mean *Root Mean Square (RMS) *fluctuational displacement
5	Solvent accessible reduction ratio
6	Partition Coefficient
7	Rigidity
8	Average surrounding hydrophobicity
9	Hydrophobicity scale (contact energy derived from 3D data)
10	Hydrophilicity scale derived from (HPLC) peptide retention data

### Features extraction

As it was mentioned earlier, we extract our physicochemical-based features from the transformed protein sequence using evolutionary information. This transformation is done using information embedded in PSSM. The transformed protein sequence is called the consensus sequence [25]. PSSM is calculated by applying PSIBLAST [26] to Gram-positive and Gram-negative data sets (using NCBI's non redundant (NR) database with its cut off value (E) set to 0.001). The PSSM consists of a *L *× 20 matrix (*L *is the length of a protein and the columns of the matrices represent 20 amino acids). It provides the substitution probability of a given amino acid with all the 20 amino acids based on its position along a protein sequence. To extract physicochemical-based features from the evolutionary consensus sequence, we first need to extract this sequence from PSSM. In the evolutionary consensus sequence, amino acids along the original protein sequence (*O*_1_, *O*_2_,...,*O_L_*) are replaced with the corresponding amino acids with the maximum substitution probability (*I*_1_, *I*_2_,...,*I_L_*). To do this, for a given amino acid, we calculate the index of the amino acid with the highest substitution probability as follows:

(1) $$I_{i}=argmax\left\{P_{ij}:1\leq j\leq 20,\;1\leq i\leq L\right\},$$

where *P_ij _*is the substitution probability of the amino acid at location *i *with the *j-th *amino acid in PSSM. We then replace the amino acid at the *i-th *location of the original protein sequence by the *j-th *amino acid to form the consensus sequence. We replace the original sequence with the consensus sequence and extract physicochemical-based features from this sequence. In this manner, we can gets benefit of evolutionary and physicochemical-based information simultaneously [17]. In the following subsections, we will first explain our proposed method to extract physicochemical-based features and then the employed methods to extract evolutionary-based features.

### Physicochemical-based features

To explore potential discriminatory information embedded in physicochemical properties of the amino acids, we extract overlapped segmented density and overlapped segmented autocorrelation feature groups.

#### Overlapped segmented density (OSD)

Global density has been widely used in protein science. It was shown that this feature provides important information of the global impact of a given attribute on the folding process [2] and is defined as follows:

(2) $$D_{global\text{\_}density}=\frac{\sum_{i=1}^{L}R_{i}}{L},$$

where *R_i _*is the attribute value (normalized) of the *i-th *amino acid. However, it fails to provide adequate local information for a given attribute [27]. In this study, we calculate local density of the amino acids using a segmentation-based technique.

**Figure 1:**
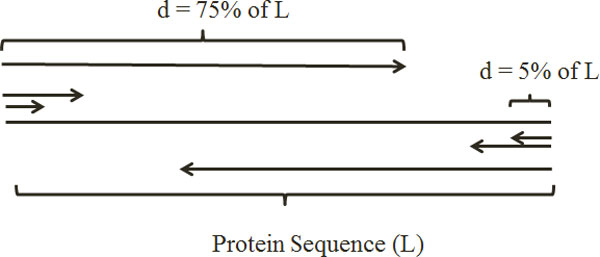
**Overlapped segmented distribution-based feature extraction method**.

In our proposed method, starting from each side of the protein sequence (left and right) we segment the protein sequence and calculate the density value for each segment. Starting from left side of the attribute sequence, we calculate local density as the sum $SD_{1}^{\left(f\right)}=R_{1}+R_{2}+...+R_{i}$ of the first *d% *(called segmentation factor) of *L *(in which the superscript *f *stands for starting from the left side of the proteins). This process is carried out for different values of *d *(5%, 10%, 15%,...,75%) to get 15 local densities $SD_{1}^{\left(f\right)},SD_{2}^{\left(f\right)},\ldots ,SD_{15}^{\left(f\right)}$. We also compute 15 features by analyzing the sequence starting from the right side of the protein sequence in the similar manner. Thus, a total of 31 features using the proposed method are extracted (1 global density + 15 from the left side + 15 from the right side). Note that we segment the protein sequence with distribution factor of *d *and process it from the left as well as from the right side of the protein sequence while the left and right side processing overlap (Figure 1). As a result, we call this method overlapped segmented distribution approach.

In this study, 5% distribution factor and 75% (called overlapping factor), are selected based on the average length of the proteins in the explored benchmarks and the experiments that were conducted by the authors [17]. The overlapping approach is proposed to provide more information about the distribution of the amino acids in the middle of a protein considering each side. Considering the number of features (only 10 overlapping features), this approach is able to provide important overlapping information to tackle this problem. This approach also enables us to explore the impact of each attribute more comprehensively compared to previously explored methods [17,27].

#### Overlapped segmented autocorrelation (OSA)

In the past the autocorrelation features have been computed using the whole protein sequence of *L *attribute values *R*_*i *_(*i *= 1,...,*L*). *Pseudo amino acid composition-based features *are good examples of these types [28]. These autocorrelation features capture the interaction of the neighboring amino acids over the entire length of the protein sequence. In the present study, we extend the concept of overlapped segmented density features as described in the previous subsection to compute the autocorrelation features from the segmented protein sequence. This is done to provide more local discriminatory information based on the interaction of the neighboring amino acids. Here we segment the protein sequence using distribution factor of 10% (*d *= 10) and overlapping factor of 70% (*o*_*f *_= 70). Using a procedure similar to the one described in the previous subsection, we first analyze the protein sequence starting from its left side and segment it for seven different values of *d *(*d *= 10%, 20%,...,70%) and calculate *DF *number of autocorrelation coefficients for each of these segments as follows:

(3) $$OSA_{i,k}=\frac{1}{\left(D_{k}^{\left(f\right)}-i\right)}\overset{D_{k}^{\left(f\right)}-i}{\underset{j=1}{\sum }}R_{j}R_{j+i},\;\left(k=1,2,\ldots ,7\;and\;i=1,\ldots ,DF\right),$$

where $D_{k}^{\left(f\right)}$ is corresponding to the number of amino acids in each segments (the number of amino acids that the summation of their physicochemical-based values is equal to $SD_{k}^{\left(f\right)}$ and *DF *is the distance factor parameter and is set to 10 as the most effective value for this parameter [17]. Note that 70 (7 × *DF *) autocorrelation coefficients are computed in this manner by analyzing the protein sequence from the left side. This process is repeated to obtain another 70 (7 × *DF *) autocorrelation coefficients by analyzing the protein sequence from the right side. We also compute the global autocorrelation coefficient of the whole protein sequence (using *DF *= 10). Thus, we have extracted a total of 150 (7 *DF *+ 7 *DF *+ *DF *= 15 × *DF *) autocorrelation features in this manner. These two physicochemical-based feature groups are extracted to provide local and global discriminatory information based on density, distribution, and autocorrelation properties simultaneously [17,25].

### Evolutionary-based features

We also extract two evolutionary-based feature groups, namely Semi-composition and Auto-covariance. These feature groups provide important evolutionary information extracted from PSSM to tackle protein subcellular localization [17].

#### Semi-composition (PSSM-SC)

This feature group is called semi-composition because we calculate the summation of the substitution probability for each amino acid from PSSM, rather than using the protein sequence directly when calculating the composition feature group. The semi-composition derived from the PSSM (consisting of 20 features) is calculated as follows:

(4) $$PSSM-SC_{j}=\frac{1}{L}\overset{L}{\underset{i=1}{\sum }}P_{i,j},\left(j=1,\ldots ,20\right).$$

#### Evolutionary-based auto covariance (PSSM-AC)

The concept of PSSM-AC has recently been used in the literature to provide more information about the interaction of the amino acids with each other along a protein sequence [17,25]. PSSM-AC gives the auto covariance of the substitution score of each amino acid with its neighboring amino acids along a protein sequence and is defined as follows:

(5) $$\begin{array}{c} PSSM-AC_{k,j}=\frac{1}{\left(L-k\right)}\overset{L-k}{\underset{i=1}{\sum }}\left(P_{i,j}-P_{ave,j}\right)\left(P_{i+k,j}-P_{ave,j}\right),\\ \;\;\;\left(j=1,\ldots ,20\;and\;k=1,\ldots ,DF\right), \end{array}$$

where *P_ave,j _*is the average substitution score of the amino acid *j *in the PSSM. A distance factor (*DF*) of 10 is used as the most effective value for this parameter [17]. Hence, there are 200 features (20 × *DF*) calculated for this feature group.

## Classification technique (Rotation Forest)

Rotation Forest is generally categorized as a Meta classifier and is based on the Random Forest classifier, Bagging, and *Principal Component Analysis (PCA) *[29]. It was introduced in [30] to enhance the performance of the Random Forest classifier by increasing the impact of diversity and individual prediction accuracy of its base learners (also called weak learners). The Rotation Forest works in the following manner. It builds independently trained decision trees to construct an ensemble of classifiers in a parallel scheme and then combines their predictions using majority voting [30]. The Rotation Forest uses a rotated feature space rather than using random subsets of features (as it is used in the Random Forest classifier) to train each base learner. To do this, the feature set of size *N *is split randomly into *K *subsets (where *K *is the number of base learners in this classifier) and then PCA is applied separately to each subset to linearly transform the feature vector. Then, by combining all *K *transformed feature subsets, a new set of *M *features is built to train each base learner [31]. Note that M is equal to N when none of the eigenvalues are zero and *M *<*N *when some of the eigenvalues are equal to zero [30,32].

As it was mentioned earlier, in the Rotation Forest classifier, the aim is to increase diversity within the ensemble classifier better than the Random Forest classifier by using the principle components [33]. This is better than the Bagging and Random Forest classifiers that use bootstrap sampling and random selection to encourage diversity [31,32,34]. Also, the individual accuracy of the base learner is considered in the Rotation Forest classifier. Unlike the Random Forest classifier, the Rotation Forest can be used with a wide range of classifiers as its base learner. Hence, it is easier to build different ensemble classifiers using the Rotation Forest classifier compared to the Random Forest classifier [30]. For this classifier, the individual accuracy is enhanced by using more accurate base learner than the Random Forest which uses naive decision tree as its base learner [30]. In this study, C4.5 decision tree is chosen because of its sensitivity to the rotation of the features, as shown by [30]. In this experiment, the data mining toolkit WEKA is used for the classification, *K *is set to 100 (as the most effective value for this parameter [31,32]) and J48 (WEKA's own version of C4.5 decision tree algorithm) was used as the base classifier.

## Results and discussion

**Figure 2:**
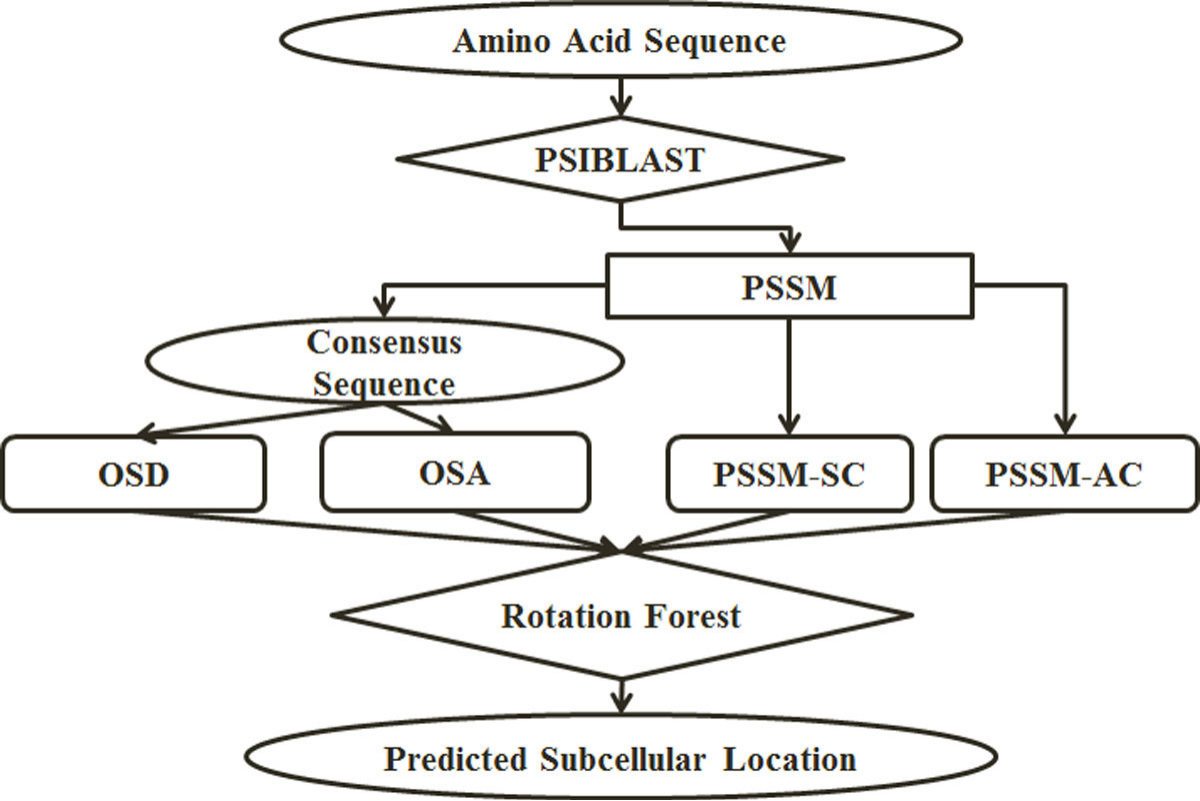
**The overall architecture of our proposed approach**.

To investigate the effectiveness of our proposed methods, we first construct a feature vector consisting of the combination of features extracted in this study. To do this, for each of the selected physicochemical attributes investigated in this study, we first extract overlapped segmented density and overlapped segmented autocorrelation feature groups. Then we combine these two feature groups with semi-composition and auto-covariance feature groups extracted from PSSM. Hence, for each physicochemical attribute, we build a feature vector consisting of 401 features (OSD(31 features), OSA (150 features), PSSM-SC (20 features), and PSSM-AC (200 features)). For the rest of this study, these feature vectors will be referred as Comb_<number> in which number refers to the number assigned to each physicochemical attribute in Table 1. The general architecture of our proposed system is shown in Figure 2.

To be able to directly compare our results with those previously reported, we use the 10-fold cross validation method. In the 10-fold cross validation method, the data set is randomly divided into 10 subsets in which in each iteration, 9 subsets are selected as training data while the remaining sub set is used as the test data. This process continues 10 times and until all the subsets are used once as the test data set [8,35]. We report our results in terms of protein subcellular prediction accuracy (in percentage) which is defined as follows:

(6) $$Q=\frac{C}{N}\times 100,$$

where *C *is the number of the correctly classified samples and *N *is the total number of samples. We apply Rotation Forest classifier to the Comb_1 to Comb_10 for our employed data sets and report the results for all the subcellular locations as well as overall prediction accuracy, as has been done in previous studies [35]. The results for Gram-positive and Gram-negative data sets are reported in Table 2 and Table 3 respectively.

**Table 2:** Results achieved for the feature vectors extracted for all 10 physicochemical-based attributes for Gram-positive data set (in percentage %) for all 4 subcellular locations ((1) Cell membrane, (2) Cell wall (3) Cytoplasm, (4) Extracellular, which are numbered from one to four respectively).

Features	(1)	(2)	(3)	(4)	Overall
Comb_1	79.9	16.7	89.9	79.7	81.6
Comb_2	78.7	16.7	89.4	80.5	81.2
Comb_3	78.7	16.7	88.9	81.3	81.2
Comb_4	81.0	11.1	88.9	79.7	81.5
Comb_5	76.4	16.6	90.7	82.1	81.5
Comb_6	78.2	16.7	89.9	80.5	81.3
Comb_7	77.0	16.7	91.3	80.5	81.5
Comb_8	77.6	16.7	90.4	82.1	81.7
Comb_9	82.2	16.7	92.3	80.5	83.6
Comb_10	79.9	16.7	91.8	82.9	83.1

**Table 3:** Results achieved for the feature vectors extracted for all 10 physicochemical-based attributes for Gram-negative data set (in percentage %) for all 8 subcellular locations ((1) Cell inner membrane, (2) Cell outer membrane, (3) Cytoplasm, (4) Extracellular, (5) Fimbrium, (6) Flagellum, (7) Nucleoid, (8) Periplasm which are numbered from one to eight respectively).

Features	(1)	(2)	(3)	(4)	(5)	(6)	(7)	(8)	Overall
Comb_1	86.9	54.0	88.5	51.1	56.3	16.7	12.5	61.7	76.4
Comb_2	86.3	50.0	88.8	50.4	59.4	25.0	00.0	58.9	75.6
Comb_3	86.7	53.2	88.3	52.6	62.5	00.0	00.0	58.9	76.0
Comb_4	87.5	56.5	87.1	49.6	59.4	00.0	12.5	62.2	76.4
Comb_5	87.4	51.6	87.3	45.9	68.8	08.3	12.5	61.7	75.9
Comb_6	86.9	52.4	86.3	52.6	68.8	00.0	25.0	62.8	76.2
Comb_7	87.0	54.0	88.3	48.9	65.6	16.7	00.0	58.9	76.0
Comb_8	86.4	55.6	87.8	51.1	59.4	08.3	25.0	61.7	76.3
Comb_9	87.7	53.2	87.6	49.6	68.8	16.7	12.5	61.7	76.6
Comb_10	86.4	53.2	87.7	51.1	65.6	08.3	12.5	60.6	75.8

As is shown in Table 2 we achieve over 81.0% prediction accuracy for all the feature vectors extracted from the physicochemical attributes explored in this study. These results are better than the 80.3% prediction accuracy reported in the literature for this task [8]. Achieving high results for all the physicochemical attributes emphasizes the effectiveness of our proposed feature extraction techniques for this task. In addition, we reach 83.6% prediction accuracy for attribute number 9 (Hydrophobicity scale (contact energy derived from 3D data)) which is better than all the other physicochemical attributes explored in this study which emphasizes the effectiveness of this attribute for protein subcellular localization. We enhance Gram-positive subcellular localization 3.3% over previously reported results found in the literature [8].

For Gram-negative data set we achieve over 75.0% prediction accuracy for all the physicochemical attributes explored in this study. These results are better than the best results reported for this data set (73.2% in [35]) which emphasizes on the effectiveness of our proposed feature extraction methods. Similarly, among the explored physicochemical attributes, using attribute number 9 (Hydrophobicity scale (contact energy derived from 3D data)) we achieve the best result. We report 76.6% prediction accuracy for Gram-negative subcellular localization which is 3.4% better than previously reported results for this data set [35]. Note that better prediction performance for Gram-positive is because of its simplicity compared to Gram-negative subcellular localization. For Gram-positive subcellular localization, the number of locations is just four and the distribution of samples in different location is more consistent. While there are eight subcellular locations for Gram-negative bacterial proteins and the number of samples in different locations is inconsistence (there are 557 and 410 samples are in the Cell inner membrane and Cytoplasm while there are 8 and 12 samples in the Flagellum and Nucleoid locations). Therefore, the prediction performance for Gram-positive is better than the prediction performance for Gram-negative which is consistent with previously reported results for these two tasks [35]. Achieving high results for both Gram-negative, and Gram-positive data sets shows the generality of our proposed methods and also preference for the Hydrophobicity scale (contact energy derived from 3D data) attribute for Gram-positive and Gram-negative protein subcellular localization. Note that for the rest of our experiments we will use this attribute (Comb_9).

In order to investigate the statistical significance of our achieved improvement for Gram-negative and Gram-positive subcellular localization prediction problems we use paired t-test. The probability value calculated for the pairwise t-test (*p *= 0.0047) emphasizes the statistical significance of our reported results and the enhancement achieved in this study.

### Impact of using Rotation Forest

To investigate the effectiveness of the Rotation Forest for protein subcellular localization, we apply the neural network that was used in [8] (back propagation ANN using *Radial Basis Function (RBF) *activation function) to our extracted features and compare the results with those achieve here. We achieve 77.4% and 71.1% prediction accuracies which are 6.2% and 5.5% less than using the Rotation Forest for Gram-positive and Gram-negative protein subcellular localization data sets, respectively. This shows the effectiveness of using the Rotation Forest which has not been explored at all for this task.

### Investigating the importance of the explored feature groups in this study

We apply the Rotation Forest to each of the explored feature groups to investigate their effectiveness on the achieved results. We then add these four feature groups together and apply the Rotation Forest to these combinations to see the impact of adding each feature group to the achieved results (using physicochemical attribute number 9). As it is shown in Table 4 combining these four feature groups together increases the prediction accuracy monotonically and the best results is achieved by combining all the feature groups together. This emphasizes the effectiveness of all the feature groups explored in this study to enhance Gram-positive and Gram-negative protein subcellular localizations.

**Table 4:** The overall prediction accuracy achieved using Rotation Forest to each feature groups investigated in this study (in percentage).

Features	Gram-positive	Gram-negative
PSSM_AAC	75.7	71.2
PSSM_AC	79.5	72.2
OSD	63.9	63.9
OSA	67.7	68.9
PSSM_AAC + PSSM_AC	80.5	74.9
PSSM_AAC + PSSM_AC + OSD	81.3	75.6
PSSM_AAC + PSSM_AC + OSD + OSA	83.6	76.6

It also shows the results achieved using Rotation Forest to the combination of feature groups to build the Combination of all for feature groups together. We use attribute number 9 (Hydrophobicity scale (contact energy derived from 3D data) attribute) for this experiment as using this attribute we attain our best results.

## Conclusion

In this study we have proposed a pattern recognition-based approach to solve Gram-positive and Gram-negative protein subcellular localizations in the following steps. First, we have investigated a wide range of physicochemical attributes using several classifiers and feature extraction techniques and selected the 10 attributes that attained the best results for protein subcellular localization. Second, using the evolutionary information embedded in PSSM, we transformed the protein sequence and also extracted semi-composition and auto-covariance feature groups directly from PSSM. Third, we extracted physicochemical-based feature groups by proposing overlapped segmented density, and overlapped segmented autocorrelation feature groups from the transformed protein sequence for all 10 physicochemical attributes mentioned earlier. Fourth, all four feature groups extracted here were combined to make a feature vector that contains both evolutionary and physicochemical discriminatory information simultaneously. Finally, by applying the Rotation Forest classifier to our extracted feature groups, we achieved 83.6% and 76.6% prediction accuracies for Gram-positive and Gram-negative subcellular localization which are 3.3% and 3.4% better than previously reported results for these two tasks, respectively [8,35].

These enhancements emphasizes the effectiveness of our proposed feature extraction techniques, the discriminatory information embedded in physicochemical-based features, and finally the Rotation Forest classifier that has not been explored for this task. For our future work, we aim at exploring wider range of feature extraction techniques to reduce the number of features as well as enhancing protein subcellular localization.

## Competing interests

The authors declare that they have no competing interests.

## Declarations

Publication of this article funded by Griffith University and National ICT Australia (NICTA).

This article has been published as part of *BMC Bioinformatics *Volume 16 Supplement 4, 2015: Selected articles from the 9th IAPR conference on Pattern Recognition in Bioinformatics. The full contents of the supplement are available online at http://www.biomedcentral.com/bmcbioinformatics/supplements/16/S4.
